# Thylakoid Rhodanese-like Protein–Ferredoxin:NADP^+^ Oxidoreductase Interaction Is Integrated into Plant Redox Homeostasis System

**DOI:** 10.3390/antiox12101838

**Published:** 2023-10-10

**Authors:** Lea Vojta, Anja Rac-Justament, Bernd Zechmann, Hrvoje Fulgosi

**Affiliations:** 1 Laboratory for Molecular Plant Biology and Biotechnology, Division of Molecular Biology, Institute Ruđer Bošković, Bijenička cesta 54, HR-10000 Zagreb, Croatia; lvojta@irb.hr (L.V.); anja.rac.justament@irb.hr (A.R.-J.); 2 Center for Microscopy and Imaging (CMI), Baylor University, One Bear Place #97046, Waco, TX 76798-7046, USA; bernd_zechmann@baylor.edu

**Keywords:** ROS, superoxide, redox homeostasis, stress response, co-immunoprecipitation, glutathione, tAPX, EPR

## Abstract

In vascular plants, the final photosynthetic electron transfer from ferredoxin (Fd) to NADP^+^ is catalyzed by the flavoenzyme ferredoxin:NADP^+^ oxidoreductase (FNR). FNR is recruited to thylakoid membranes via an integral membrane protein TROL (thylakoid rhodanese-like protein) and the membrane associated protein Tic62. We have previously demonstrated that the absence of TROL triggers a very efficient superoxide (O_2_^•−^) removal mechanism. The dynamic TROL–FNR interaction has been shown to be an apparently overlooked mechanism that maintains linear electron flow before alternative pathway(s) is(are) activated. In this work, we aimed to further test our hypothesis that the FNR–TROL pair could be the source element that triggers various downstream networks of chloroplast ROS scavenging. Tandem affinity purification followed by the MS analysis confirmed the TROL–FNR interaction and revealed possible interaction of TROL with the thylakoid form of the enzyme ascorbate peroxidase (tAPX), which catalyzes the H_2_O_2_-dependent oxidation of ascorbate and is, therefore, the crucial component of the redox homeostasis system in plants. Further, EPR analyses using superoxide spin trap DMPO showed that, in comparison with the wild type, plants overexpressing TROL (TROL OX) propagate more O_2_^•−^ when exposed to high light stress. This indicates an increased sensitivity to oxidative stress in conditions when there is an excess of membrane-bound FNR and less free FNR is found in the stroma. Finally, immunohistochemical analyses of glutathione in different Arabidopsis leaf cell compartments showed highly elevated glutathione levels in TROL OX, indicating an increased demand for this ROS scavenger in these plants, likely needed to prevent the damage of important cellular components caused by reactive oxygen species.

## 1. Introduction

Thylakoid rhodanese-like protein (TROL) is an integral thylakoidal membrane protein that serves as a docking site for the important photosynthetic enzyme ferredoxin:NADP^+^ oxidoreductase (FNR). Besides its role in photosynthetic energy conversion, FNR plays an important role in redox poising of both thylakoids and stroma. In plants and bacteria, FNR is also an important scavenger of free radicals [1]. TROL is built out of several structurally and functionally different regions: the N-terminal stromal domain including chloroplast targeting pre-sequence [2], the two transmembrane domains, the rhodanese-like domain (RHO) in thylakoid lumen, and the C-terminal domain protruding into the stroma [3]. The C-terminal stretch comprises of the proline-rich region PEPE that provides flexibility and swiveling motion, and the very terminal ITEP domain which is responsible for protein–protein recognition and the interaction with the FNR dimer [2,3,4]. It has been shown that the TROL–FNR binding is dynamic [5], light-dependent [5], and influenced by chloroplast energetic needs—prioritizing either energy production or energy dissipation [6]. When bound to the TROL, FNR primarily reduces NADP^+^, creating chemical energy equivalents necessary to fulfill the needs of the cell for complex sugars which are synthetized by the process called the Calvin–Benson Cycle, as well as the downstream processes. The free FNR, on the other hand, has the ability to engage in excess energy dissipation and the removal of reactive oxygen species (ROS) after exposure to environmental stresses [6]. For instance, we have previously demonstrated that Arabidopsis plants that do not accumulate TROL (TROL knock-out, KO, *trol*) have the ability of enhanced and very rapid removal of superoxide anion (O_2_^•−^), produced in chloroplasts either physiologically or by external induction [6].

Benz et al. proposed the model for FNR binding to plant thylakoids in which the majority of chloroplast FNR is bound to thylakoids via Tic62 and TROL proteins during periods of darkness, presumably, in that way stabilizing the FNR enzyme in the hours of photosynthetic inactivity [7]. When light conditions change, such as in the morning hours, FNR is being released to stroma, where it acts as an efficient NADPH catalyst, allowing efficient linear electron transfer (LET) [7,8]. Contrary to this scenario, Forti and Bracale [9] reported that NADP^+^ photoreduction is very inefficient when the enzyme is not bound to the membranes. Hanke et al. [10] reported that thylakoids devoid of FNR could not reproduce WT rates of NADPH production, which cannot be overcome, even with the addition of high concentrations of soluble FNR. We have proposed the dynamic FNR recruitment to TROL [11], in which the TROL-bound FNR performs efficient NADP^+^ photoreduction in normal light conditions. This is due to its recruitment at the vicinity of photosystem I (PSI) which is necessary for the directed electron transfer from the reduced Fd. In the high-light conditions, FNR detaches from TROL, probably by the transmembrane signaling involving RHO-like and PEPE domains of the protein. Released FNR can act as an efficient ROS scavenger, or Fd can distribute electrons to various other acceptors. We have postulated that the TROL–FNR pair could be the source element in the signal transduction cascade linking photosynthesis with plant growth and cellular responses. Moreover, it contains several elaborate elements of signal transduction: the luminal RHO-like domain, the proline-rich swivel involved in the signal attenuation, and the FNR membrane recruitment region [11].

In chloroplasts, O_2_^•−^ evolves at the level of photosystem I (PSI) even under conditions that are favorable for photosynthesis [12]. The enzyme superoxide dismutase (SOD) catalyzes the transition of O_2_^•−^ to H_2_O_2_ and O_2_. H_2_O_2_ is further scavenged by ascorbate peroxidase (APX). The primary product of oxidation in the APX-catalyzed reaction, the monodehydroascorbate radical, is photoreduced to ascorbate in the reaction mediated by Fd [13]. Chloroplastic APX can be found in thylakoid bound (tAPX) and stroma-localized forms. tAPX binds in the vicinity of PSI [14,15], and, except in Arabidopsis, a single gene encodes both tAPX and the stroma localized APX, while both isoforms are generated by alternative splicing [16]. APXs play an important role in the redox homeostasis system of plants by reducing H_2_O_2_ to water [17]. In chloroplasts, most of Cu/Zn-SOD attaches to the stroma thylakoids close to PSI [12]. PSI-attached SOD, tAPX bound to thylakoids in the vicinity of PSI, and the Fd-dependent reduction of monodehydroascorbate radical (MDA), together form the thylakoidal scavenging system and function as the first defense against ROS [18]. Other scavenging enzymes are located in the stroma and represent the second defense against ROS (stromal scavenging system). It has been shown that the cooperation of chloroplast ascorbate peroxidases and the proton gradient regulation 5 (PGR5) is critical for protecting Arabidopsis plants from photooxidative (high light) stress [19]. In addition, it was proposed that by controlling H_2_O_2_ availability, chloroplast APXs can act as regulators of chloroplast-to-nucleus (retrograde) signaling. It was shown that their overexpression suppresses the HL-induced expression of H_2_O_2_-responsive genes [20,21].

In plants, the glutathione–ascorbate cycle (Foyer–Halliwell–Asada pathway) operates in the cytosol, mitochondria, plastids, and peroxisomes [22,23]. It is believed that the glutathione–ascorbate cycle plays the key role in H_2_O_2_ detoxification, since glutathione, ascorbate, and NADPH are present in high concentrations in plant cells. However, enzymes like peroxiredoxins and glutathione peroxidases, which use thioredoxins or glutaredoxins as reducing substrates, and contribute to H_2_O_2_ removal in plants as well [24].

In this work, we addressed the proposed role of TROL in plant redox homeostasis by investigating its interaction with crucial components of the oxidative stress response in plants, such as APX and SOD. Furthermore, in TROL knock-out (KO, *trol*) and the TROL overexpression (TROL OX) Arabidopsis lines, we investigated the quantity of glutathione (GSH) as the preventer of possible cellular damage caused by the ROS [25]. By using electron paramagnetic resonance (EPR) we have previously measured the superoxide radical detoxification rates of the wild type and the TROL KO chloroplasts and have determined that the chloroplasts from the TROL KO plants which have been pre-acclimated to different light conditions consistently exhibit diminished O_2_^•−^ accumulation [6]. And, perhaps even more interesting, the dark and the growth-light-acclimated *trol* chloroplasts were resilient to O_2_^•−^ hyper-propagation induced by the methyl viologen [6]. Finally, we further tested our hypothesis by asking what happens to superoxide anion propagation when there is an overproduction of TROL (like in TROL OX line) and when predictably less free FNR can be found in stroma.

## 2. Materials and Methods

### 2.1. Plant Material and Growth Conditions

*Arabidopsis thaliana* (L.) ecotype Columbia (Col-0) plants (originally obtained from the European Arabidopsis stock centre, NASC, Loughborough, UK) were grown in the growth chamber (Kambič, Slovenia) on the sieved growth substrate A400 (Stender, Germany) under the light intensity of photosynthetically active radiation (PAR) 80 μmol photons m^−2^·s^−1^ (Osram Flora, Osram, Germany), and 16 h/8 h light/dark period at 22 °C, relative humidity 60% during the day and 70% during the night. TROL (*At4g01050*) overexpressing mutant line (TROL OX) was grown under the same conditions, except the light intensity of PAR 20 μmol photons m^−2^·s^−1^ was applied, since these plants are very photosensitive and do not grow well in higher light intensities. To construct TROL overexpression line, the *trol* knock-out *A. thaliana* plants [2] were transformed by using plasmid vector pH7WG2.0 (35S promoter) containing TROL-HA-FLAG construct, in which both peptide tags were added at the very C-terminus [26]. 

### 2.2. Chemicals

The spin trap 5,5-dimethyl-1-pyrroline-N-oxide (DMPO) and N, N′-dimethyl-4,4′-bipyridinium dichloride (Methyl viologen dichloride hydrate, MV) were purchased from the Sigma Chemical Co. (Aldrich Corp., St. Louis, MO, USA).

### 2.3. Chloroplast Isolation from A. thaliana 

Intact Arabidopsis chloroplasts were isolated from 4 week old plants as described by Jurić et al. [2]. After final centrifugation for 5 min at 4 °C at 1500× *g*, pellet was resuspended in the buffer (330 mM Sorbitol and 20 mM Tris/HCl pH 8.4) and chloroplasts were purified on an 80% Percoll cushion by centrifugation for 10 min at 6000× *g* and 4 °C. Chloroplast concentration was set to 1 mg chlorophyll per 1 mL buffer. 

### 2.4. EPR Measurements

Two days before EPR measurement, plants were adapted either to dark (D), for the wild-type, or to the growth-light (GL) PAR 80 μmol photons m^−2^·s^−1^,and for the TROL OX line to the growth-light PAR 20 μmol photons m^−2^·s^−1^, or to the high-light (HL) PAR 350 μmol photons m^−2^·s^−1^, with 16/8 light/dark periods. The formation of ROS was measured with the EPR spectroscopy by using the specific superoxide anion spin trap molecule DMPO in the reaction mixture containing chloroplasts equivalent to 50 μg of chlorophyll supplemented with the 433 mM DMPO. In the experiments with the MV, its final concentration in the mixture was 10 mM. Immediately after the addition of the spin trap, the samples in the glass capillaries (inner diameter of 1 mm) were, for 30 s, either illuminated with the PAR of 100 μmol photons m^−2^·s^−1^ or were kept in the dark. Measurements of the ROS formation were performed on an X-band Varian E-109 spectrometer by using the following instrumental set-up: microwave power of 20 mW, modulation amplitude of 0.1 mT, modulation frequency of 100 kHz, and scan range of 8 mT. All measurements were performed at room temperature and the data were collected by using the manufacturers’ software. In both DMPO and MV measurements, the data related to the TROL OX chloroplasts were normalized with respect to the WT data, which were taken as the reference representing 100% radical yield (fold value of 1).

### 2.5. Tandem Affinity Purification and MS Analysis

A total of 50 g of fresh 5-week-old Arabidopsis TROL OX plants were used for chloroplast isolation. To isolate thylakoid membranes, chloroplasts corresponding to approximately 25 mg of chlorophyll were lysed in the dark in 10 mM Tris/HCl pH 6.8, 10 mM MgCl_2_ and 20 mM KCl for 5 min on ice, pelleted, washed twice in the same buffer, and solubilized in solution containing 750 mM *χ*-aminocapronic acid, 50 mM Bis-Tris, 0.5 mM EDTA, and 1% dodecyl maltoside (DDM) (*w*/*v*) for 30 min on ice. After the centrifugation at 21,000× *g* 10 min at 4 °C, supernatant was diluted 3 times in the solubilization buffer without DDM and with the addition of 1 tablet of protease inhibitors (Complete ULTRA EDTA free tablet, Roche, Basel, Switzerland). Then, 150 µL of the ANTI-FLAG slurry (FLAG HA Tandem Affinity Purification Kit, Sigma-Aldrich, St. Louis, MO, USA) was added into the sample and incubated under rotation (12 rpm) at 4 °C, overnight. On the next day, the sample was pelleted by the centrifugation at 1000× *g* 2 min at 4 °C and washed with the 750 mM *χ*-aminocapronic acid, 50 mM Bis-Tris, and 0.5 mM EDTA buffer. After centrifugation at 1000× *g* for 2 min at 4 °C, the matrix was washed in a RIPA buffer containing protease inhibitors, according to the manufacturer protocol. The sample that remained bound to the anti-FLAG resin was eluted by using 3 × FLAG peptide in TBS buffer. To the eluate, 30 µL of ANTI-HA slurry (FLAG HA Tandem Affinity Purification Kit, Sigma-Aldrich) was added and incubated under rotation for 1.5 h at 4 °C. After washing the matrix 3 times with TBS buffer, the sample bound to the anti-HA resin was eluted by using 8 M Urea and frozen immediately after elution. Additionally, the sample remaining bound to the HA-matrix was eluted by incubation in a Laemmli buffer [27] at 85 °C for 5 min. The samples were shipped on dry ice to the MS facility (MS service, MPI CBG, Dresden, Germany), where they were analyzed by one-dimensional sodium dodecyl sulfate-polyacrylamide gel electrophoresis followed by liquid chromatography-tandem mass spectrometry (GeLC-MS/MS). Prior to the MS analysis, the small aliquot of eluate was tested by the Western blot to check for the presence of the bait. Tandem Affinity Purification and mass spectrometry were performed three times, independently. Only proteins found to interact with TROL in all three trials with a protein threshold of 99.9% and peptide threshold of 95% were further considered.

### 2.6. Co-Immunoprecipitation

Intact chloroplasts were isolated from 5-week-old Arabidopsis WT or TROL OX plants, as described previously [6], and lysed for 5 min on ice in 10 mM Tris/HCl pH 6.8, 10 mM MgCl_2_, and 20 mM KCl solution. Membranes were pelleted at 1500× *g* for 5 min at 4 °C, followed by solubilization in 750 mM *χ*-aminocaproic acid, 50 mM Bis-Tris, 0.5 mM EDTA and 1% DDM (*w*/*v*). Solubilization was performed for 30 min on ice, in the dark. After centrifugation at 21,000× *g* for 10 min at 4 °C, the supernatant was diluted 3 times in the solubilization buffer without DDM and divided into four parts for incubation, with either 50 µL preimmune serum or 50 µL anti-TROL (Agrisera AS194257) and an addition of 150 mM NaCl, or without salt. In all reaction mixtures, 1 tablet of protease inhibitors (Complete ULTRA EDTA free tablet, Roche, Basel, Switzerland) was added and mixtures were rotated at 12 rpm at 4 °C, overnight. On the next day, 30 µL of ProteinA Agarose slurry (NEB, Ipswich, MA, USA) was added into each immunoprecipitation mixture and incubated at 12 rpm at RT, for 90 min. Samples were centrifuged at 1000× *g* for 4 min and beads were washed 3 times in 750 mM *χ*-aminocaproic acid, 50 mM Bis-Tris, and 0.5 mM EDTA buffer. After the last wash, the pellet (beads) was vortexed in Laemmli buffer [27] and shortly spun down. The procedure was repeated, and the resulting two fractions were combined into the eluate. Samples for further analysis, taken after each incubation or purification step, were separated by the SDS-PAGE on 12% gels, and analyzed by Western blot at 200 mA for 1 h 15 min. Anti-APX (1:2000, Agrisera AS08368, 1:1000 PhytoAB PHY1322A), anti-CSD2 (Cu/Zn chloroplastic SOD, 1:1000, Agrisera AS06170) sera were used for overnight incubation with agitation at 4 °C. As the secondary antibody, anti-rabbit IgG peroxidase conjugate (1:10,000) was incubated with the membranes for 1 h 15 min at RT. The results were visualized by the ECL on X-ray film.

### 2.7. Plant Material and Growth Conditions for Glutathione Determination

After stratification (2 days at 4 °C), seeds of *Arabidopsis thaliana* [L.] Heynh. ecotype Columbia (Col-0), the TROL overexpressing (OX), and the TROL knock-out (KO) lines were grown on substrates in growth chambers with 10 h/14 h day/night photoperiod and relative humidity of 60%. Day and night temperatures were 22 °C and 18 °C, respectively, and plants were kept at 100% relative soil water content under light intensity of PAR 80 µmol photons m^−2^·s^−1^. Four weeks after stratification and 4 h after the onset of the light period, samples from the youngest fully developed rosette leaf were harvested and prepared for electron microscopy. Leaves at this stage were approximately 2 cm long and 0.7 cm wide. Exposure of 4-week-old plants to HL occurred at PAR 350 µmol photons m^−2^·s^−1^ for 4 h in the same growth conditions as described above. All mentioned growth conditions were at the Institute of Plant Sciences, Graz, Austria.

### 2.8. Sample Preparation for Transmission Electron Microscopy and Immunogold Labeling

Preparation of samples for transmission electron microscopy and immunogold labeling of glutathione was performed with ultrathin sections on nickel grids as described in [28]. Small samples of the youngest fully developed leaves (about 1.5 mm^2^) from at least three different plants were fixed in 2.5% paraformaldehyde/0.5% glutardialdehyde in 0.06 M phosphate buffer (pH 7.2) for 90 min at RT. After fixation, samples were washed in 0.06 M phosphate buffer (pH 7.2) and dehydrated in increasing concentrations of acetone (50%, 70%, and 90%) at RT, repeated 2 times for 10 min for each step. Subsequently, specimens were gradually infiltrated with increasing concentrations of LR White resin (30%, 60%, and 100%; London Resin Company Ltd., Berkshire, UK), mixed with acetone (90%), and finally embedded in LR White resin and polymerized for 48 h at 50 °C in small plastic containers. Reichert Ultracut S ultramicrotome (Leica Microsystems, Vienna, Austria) was used for cutting ultrathin sections (80 nm) of the samples.

### 2.9. Cytohistochemical Determination of Glutathione

Immunogold labeling of glutathione was performed with ultrathin sections on coated nickel grids with the automated immunogold labeling system Leica EM IGL (Leica Microsystems, Vienna, Austria) according to [28]. For cytohistochemical analysis, samples were blocked with 2% bovine serum albumin (BSA) in phosphate-buffered saline (PBS, pH 7.2) and then treated with the primary antibody (anti-glutathione rabbit polyclonal IgG; Millipore Corp., Billerica, MA, USA) and diluted 1:50 in PBS containing 1% goat serum for 2 h at RT. After a short rinse in PBS (three times for 5 min), 10 nm gold-conjugated secondary antibody (goat anti-rabbit IgG, British BioCell International, Cardiff, UK) diluted 1:50 in PBS was added to the samples and incubated at RT for 90 min. After incubation, samples were briefly washed in PBS (3 × 5 min) and distilled water (2 × 5 min). Labeled grids were either immediately observed in the Philips CM10 transmission electron microscope or post-stained with uranyl-acetate (2% dissolved in aqua bidest) for 15 s. Post-staining with uranyl acetate facilitates the distinction of different cell structures enabling clearer identification of the organelles of interest. The specificity of the immunogold labeling procedure was tested by several negative controls which were treated either with: (i) pre-immune serum instead of the primary antibody; (ii) gold-conjugated secondary antibody (goat anti rabbit IgG) without the primary antibody; (iii) non-specific secondary antibody (goat anti mouse IgG); and (iv) primary antibodies pre-adsorbed with an excess of glutathione for 2 h at RT prior to labeling of the sections. For the latter, the solution containing 10 mM of glutathione (GSH or GSSG) was incubated with 0.5% glutardialdehyde for 1 h. Incubation for 30 min in the solution of 1% (*w*/*v*) BSA saturated the excess of glutardialdehyde. The resulting solution was used to saturate the glutathione antibody for 2 h prior to its use in the immunogold labeling procedure described above.

### 2.10. Quantitative Analysis of Immunogold Labeling

Micrographs of randomly photographed immunogold-labeled sections were digitized, and gold particles were automatically counted by using the particle analysis tool of the software package *Cell D* (Olympus, Life and Material Science Europa GmbH, Hamburg, Germany) in different visually identified cell structures (mitochondria, plastids, nuclei, peroxisomes, cytosol). Due to the low detected amount of gold particles in cell walls, ER, and dictyosomes, no statistical evaluation of the gold particle density was made for these compartments. We examined at least three different samples for statistical evaluation. Gathered data are presented as the number of gold particles per µm^2^ and include a minimum of 20 (peroxisomes) to 60 (other cell structures) sectioned cell structures of at least 15 different cells throughout the blocks. Unspecific background labeling was determined for 30 different sections (outside the specimen) from 5 different samples and subtracted from the values obtained in the sample. Unspecific background labeling was around 0.3 gold particles per µm^2^. Statistical analyses included non-parametric Kruskal–Wallis test followed by a post hoc comparison according to Conover [29]; *p* < 0.05 was considered as significant.

## 3. Results

### 3.1. TROL May Form Transient Complexes with the tAPX

In the search for TROL interaction partners, affinity purification using a combination of anti-FLAG and anti-HA matrixes (Tandem Affinity Purification, TAP) of fresh chloroplast extract has been performed. For this purpose, Arabidopsis plants overexpressing protein TROL (TROL OX line) have been used. Since the TROL OX line expresses the protein TROL labelled with the HA and FLAG peptide epitopes on its C-terminus, highly purified TROL interaction complexes were obtained by using TAP. Purified complexes were further separated by SDS-PAGE and subsequently analyzed by liquid chromatography-tandem mass spectrometry (GeLC-MS/MS, Table 1). Three independent TAP/MS analyses have been performed and only proteins found to interact with the TROL in all three trials with a protein threshold of 99.9% and peptide threshold of 95% were further considered (Table 1). As anticipated, the interactions with both chloroplastic ferredoxin:NADP^+^ oxidoreductase leaf isoenzyme 1 (FNR1, At5g66190) and the isoenzyme 2 (FNR2, At1g20020) have been confirmed. Besides various components of the photosynthetic apparatus (Table 1), we have found the association of the TROL with the thylakoidal ascorbate peroxidase (tAPX, At1g77490) interesting, and have thus attempted to investigate the interaction with this ROS-scavenging enzyme in more detail. 

To test the MS results we have performed the series of co-immunoprecipitation experiments by using Arabidopsis wild-type and the TROL OX chloroplasts and the anti-TROL antiserum as the bait (the pre-immune serum was used in the control experiment). We have chosen the TROL OX line in order to provide the same experimental conditions as used for the TAP and the MS analyses. In addition, in the wild type, the amount of protein TROL was lower than in the TROL OX line, making it harder to isolate and visualize interaction complexes. However, we have also used the WT plants to test selected protein interactors in the non-modified Arabidopsis. Complexes containing TROL were pulled out from the mixture by using Protein A agarose matrix (New England Biolabs, Ipswich, MA, USA), and the samples were further separated by SDS-PAGE on 12 % gels, and finally analyzed by Western. Membranes were probed by using anti-APX and anti-CSD2. Although with MS in the TAP extracts, we did not find SOD; we have nevertheless used the anti-CSD2 (antibody against plant SOD) to investigate possible interaction of TROL with this important ROS-scavenging enzyme. SOD catalyzes the dismutation (or partitioning) of the superoxide radical into ordinary molecular oxygen and hydrogen peroxide and is therefore part of the plant redox homeostasis system [30]. Coimmunoprecipitation experiments failed to confirm the APX and SOD associations with the TROL, although, we have used several primary antibodies from different producers (please see Section 2.6). However, they all showed to be quite unspecific with very weak reactivity. Therefore, with this experimental approach we can neither confirm nor exclude the existence of such complexes.

### 3.2. TROL OX Arabidopsis Chloroplasts Accumulate Elevated Levels of O_2_^•−^ during the Exposure to High Light

**Figure 1:**
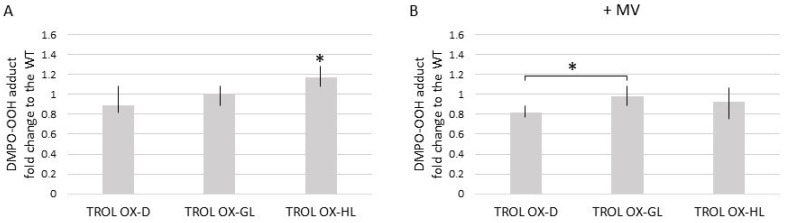
The EPR study of DMPO-OOH spin adduct formation in chloroplasts isolated from Arabidopsis TROL OX plants acclimated to different light conditions. Prior to the EPR measurements plants were kept for 48 h in dark (D), or under growth-light (GL) (PAR 80 μmol photons m^−2^·s^−1^ for WT or 20 μmol photons m^−2^·s^−1^ for OX), or high-light (HL) (PAR 350 μmol hν m^−2^·s^−1^) conditions with the photoperiod 16 h light/8 h dark. For measurement, immediately after the addition of the spin-trap, isolated chloroplasts were illuminated (PAR 100 μmol hν m^−2^·s^−1^) for 30 s. (**A**) The superoxide anion production in TROL OX chloroplasts is represented as the fold change to the WT chloroplasts; (**B**) the superoxide anion production in TROL OX chloroplasts incubated with the MV, represented as the fold change to the WT chloroplasts treated under the same conditions. Data were analyzed by one-way ANOVA and Student’s Two Sample *t*-Test. Data means of five independent measurements with error bars representing minimum and maximum values are shown. Asterisks indicate experimental data that are significantly different (*p* < 0.05).

To further investigate the connection between TROL and the plant oxidative stress responses, we have performed electron paramagnetic resonance (EPR) experiments on the TROL OX Arabidopsis line. We have used superoxide (O_2_^•−^) specific spin trap DMPO (5,5-dimethyl-1-pyrroline-N-oxide) to detect the propagation of O_2_^•−^ in conditions when FNR docking partner TROL is overexpressed (in contrast to the previous experiments on the TROL KO line). As reported previously, by using different light acclimation conditions we wanted to induce interactions of FNR with different complexes or membrane domains. Illuminated isolated intact Arabidopsis WT chloroplasts incubated with the DMPO showed a dominant DMPO-OOH spin adduct formation typical of the superoxide radical propagation, according to the EPR spectral parameters. The apparent hyperfine splitting constants of DMPO-OOH (aN = 1.42 mT, aHβ = 1.14 mT, aHγ = 0.115 mT) are in line with the published literature [31]. In the presence of SOD, the corresponding EPR signal was suppressed [6,32]. As the lifetime of the superoxide anion is short with respect to the time scale of the EPR experiment, the superoxide anion “end-product”, in terms of DMPO-OH hydroxyl radical spin adduct, is detected as well (aN = 1.49 mT, aH = 1.47 mT). Immediately after the addition of the spin trap, samples were either kept in the dark or illuminated with photosynthetic light of 100 μmol photons m^−2^·s^−1^ for 30 s. The analysis of DMPO-OOH spin adduct formation in chloroplasts from TROL OX plants acclimated to the dark revealed no significant differences in the EPR signal intensity with respect to the WT plants (Figure 1A). In growth light conditions, superoxide formation in TROL OX chloroplasts does not differ from the WT plants. However, in high light, significantly more DMPO-OOH spin adduct was observed in TROL OX (Figure 1A). When the EPR data measured for the TROL OX chloroplasts were normalized to the WT data, which are assumed to represent the 100% radical yield, around a 20% increase in high-light dependent generation of superoxide anion could be detected (Figure 1A). These findings imply that, in the excess of TROL, photo-generated electrons easily spill over to O_2_, or that the O_2_^•−^ is clearly less efficiently scavenged. 

To investigate the DMPO-OOH spin adduct formation in intensive O_2_^•−^ production conditions, we have incubated chloroplasts with the herbicide methyl-viologen (MV). In illuminated chloroplasts, MV massivelytraps electrons from the PSI, specifically from the reduced Fd to form its cation radicals, rapidly generating O_2_^•−^ via auto oxidation [33]. EPR spectroscopy detected greater DMPO-OOH spin adduct formation in the samples exposed to MV, in comparison to the non-exposed ones. When the experimental data of radical production in WT chloroplasts exposed to MV were taken as referent values representing 100% of O_2_^•−^ yield (Figure 1B), it could clearly be noticed that a significant difference was detected only in the dark-adapted TROL OX chloroplasts, where around 20% less DMPO-OOH spin adduct formation relative to the WT was observed. High-light-adapted TROL OX chloroplasts showed similar O_2_^•−^ production to the WT (Figure 1B).

### 3.3. Glutathione Levels Are Elevated in All Cell Compartments in TROL OX Plants after Short-Term Light Stress

**Figure 2:**
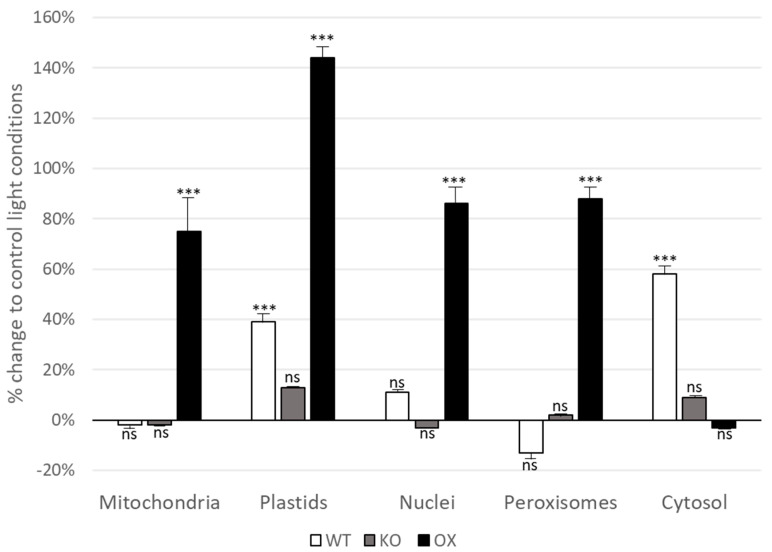
Compartment specific changes in glutathione labeling density after short-term light stress. Graphs show the percentage of increase and decrease in gold particles bound to glutathione per µm^2^ in mesophyll cells of *Arabidopsis thaliana* Col-0 plants (white bar), Arabidopsis TROL knock-out mutant (KO, grey bars), and the Arabidopsis TROL overexpression line (OX, black bars) after the exposure to a PAR intensity of 350 µmol hν m^−2^·s^−1^ for 4 h when compared to the respective plant line grown at PAR 80 µmol hν m^−2^·s^−1^. *n* > 20 for peroxisomes and *n* > 60 for other cell structures. Data means are with standard errors. Significant differences were calculated using the Mann–Whitney U-test; *** indicates significance at the 0.001 levels of confidence. ns = not statistically different.

To further investigate the role of TROL in antioxidant plant protection, we used transmission electron microscopy and immunogold labeling to determine the amounts of glutathione in different Arabidopsis compartments in WT, KO, and TROL OX plants. Control plants were grown under PAR 80 µmol hν m^−2^·s^−1^ for four weeks, while the stressed plants were exposed to high-light growth conditions of PAR 350 µmol hν m^−2^·s^−1^ light for 4 h before sampling. Immunogold labeling of glutathione was performed on ultrathin leaf sections on coated nickel grids using the anti-glutathione rabbit polyclonal IgG antibody. The antibody does not discriminate between reduced and oxidized glutathione; therefore, our results represent the total GSH in analyzed compartments. Figure 2 shows the percentage of increase and decrease in gold particles bound to glutathione per µm^2^ in mesophyll cells of *Arabidopsis thaliana* WT, KO, and TROL OX lines after the exposure to a light intensity of 350 µmol hν m^−2^·s^−1^ for 4 h, as compared to the respective plant line grown at 80 µmol hν m^−2^·s^−1^. TROL OX plants showed significant elevation in GSH content in all tested compartments except in the cytosol. In chloroplasts, 140% GSH was found in high-light stressed TROL OX plants, as compared to non-stressed plants (Figure 2). 

In mitochondria, nuclei, and peroxisome this number was elevated to around 75%, 85%, and almost 90%, respectively, as compared to the physiological (non-stressed conditions). Wild-type plants did not show significant change in light-stress-induced GSH content except in plastids, where we found 40% more GSH, and in the cytosol, with 60% more GSH than under the growth-light (Figure 2). KO plants did not show any significant change in GSH content under light stress in any of the tested compartments.

## 4. Discussion

In our previous research we have shown that TROL KO plants exhibit increased ability of photosynthetic machinery to dissipate excess energy [2]. TROL KO has greatly reduced amounts of thylakoid-bound FNR, as compared with the wild-type Arabidopsis. Additionally, genes encoding proteins involved in stress management were strongly upregulated [2]. When exposed to light intensities of PAR 800 μmol photons m^−2^·s^−1^ for two days, we observed distinctive phenotype of TROL KO plants; namely that TROL KO plants remained green, while the WT accumulated anthocyanins and initiated flowering, both signs of photooxidative stress [6]. Isolated intact chloroplasts from TROL KO plants, pre-acclimated to different light conditions, consistently manifested diminished O_2_^•−^ accumulation, showing that the absence of TROL triggers a very efficient O_2_^•−^ scavenging mechanism(s). We proposed that the dynamic binding and release of FNR from TROL represents a novel and efficient mechanism that maintains LET before pseudo-cyclic flow is activated. Accordingly, the FNR–TROL branch point could be the source element that modulates various downstream networks of plant redox homeostasis system [6]. Protein–protein interactions investigated in this work further elucidate the role of TROL protein in chloroplast stress response(s). 

By using TAP, we have detected several proteins found in the complex with the TROL, either interacting (like previously confirmed FNR [2,6,7,8,34]), or with still unknown relations to the TROL. Among some PSI components (like Chlorophyl a-b binding proteins, Table 1), tAPX has been detected during GeLC-MS/MS analysis of TAP eluates. This enzyme has been found bound in the vicinity of PSI, where, together with Cu/Zn SOD, forms the thylakoidal scavenging system—the first defense against ROS [18]. The Mehler reaction photoreduces O_2_ to O_2_^•−^ downstream PSI in the so-called water–water cycle. The superoxide is subsequently scavenged as water by SOD [35] and APX. APX is a member of the family of heme-containing peroxidases and catalyzes the H_2_O_2_-dependent oxidation of ascorbate in plants, algae, and certain cyanobacteria [36], therefore, being a crucial component of the oxidative stress response in plants. The APX enzyme functions as a linking molecule for maintaining the redox balance under stress by interconnecting two pathways, ascorbate and glutathione [37], and since both pathways in chloroplasts were found to be associated with NADPH [38,39], APX-ascorbate also regulates the NADP^+^/NADPH ratio under stress conditions. The cooperation of APX-ascorbate and glutathione is known as the Ascorbate–Glutathione (ASC-GSH) pathway or Foyer–Halliwell–Asada pathway. It functions in cytosol, chloroplast, mitochondria, and peroxisomes in both plants and animals [40,41]. The ASC-GSH pathway is known as the center of the redox homeostasis and performs the function of a consolidated scavenger of ROS [30,37]. As we have hypothesized in our previous works, FNR–TROL bifurcation could be the source element that modulates various downstream networks of plant ROS detoxification [6]. The possible interaction of TROL protein with APX suggests its role in this plant redox homeostasis system.

Although co-immunoprecipitation experiments failed to re-confirm the TROL–APX interaction, or the association with the Cu/Zn-SOD (CSD2), we cannot exclude the existence of such complexes. All primary antibodies against tAPX and SOD available on the market were tested and they were all shown to be very weak and/or unspecific. The characteristic signal was visible only in total plant extracts, when using a large amount of the sample. In the same conditions in which the antibody’s targets were detectable, the background was too strong to detect anything with certainty. Both WT and TROL OX Arabidopsis lines have been used in these experiments. We have used the TROL OX line to provide the same experimental setup as for the tandem affinity purification and the MS analyses, since we tested the possible interactions indicated by MS results. The WT was used as a kind of control to see the situation in non-manipulated plants. In general, in WT plants the amount of the protein TROL is significantly lower than in the TROL OX line, making it harder to isolate and visualize interaction complexes. Furthermore, plants in these experiments were not exposed to any kind of stress during their growth and development, which minimizes the occurrence of the production and activity of oxidative stress proteins, such as APX and SOD. We presume that such complexes might exist but are most likely transient and can hardly be detected under normal growth conditions. 

To further test the hypothesis that the release of FNR from TROL triggers an efficient O_2_^•−^ scavenging mechanism, EPR experiments were performed on the TROL OX Arabidopsis line. Opposite to the results from the TROL KO, significantly more DMPO-OOH spin adducts in TROL OX chloroplasts grown under HL conditions were detected (Figure 1), indicating that Arabidopsis plants overproducing TROL protein fail to successfully cope with the challenging environmental conditions that enhance ROS production. These results are in accordance with the previously investigated superoxide removal potential of the TROL KO, which was shown to be extraordinary, especially in the presence of methyl-viologen, the herbicide that propagates vast amounts of O_2_^•−^ [6]. In TROL OX, however, we did not observe any significant difference in O_2_^•−^ anion scavenging rates when methyl-viologen was added, except for in the dark-grown plants. It appears that, unlike the TROL KO line, the TROL OX line does not provide an efficient O_2_^•−^ anion scavenging potential. Therefore, we propose that in the excess of TROL more FNR can be found in the membrane-bound state, with less FNR being available in the stroma for ROS scavenging. Bound FNR primarily reduces NADP^+^ that is needed for the Calvin–Benson Cycle and production of carbohydrates, while free FNR transfers electrons to other acceptors in the stroma, like the ones involved in abiotic stress prevention. 

GSH is a tripeptide antioxidant found in plants, animals, fungi, and some bacteria and archaea. It possesses a capacity to prevent damage to important cellular components caused by reactive oxygen species such as free radicals, peroxides, lipid peroxides, and heavy metals. GSH neutralizes them by reducing them. By determining the glutathione content in different Arabidopsis compartments in WT, TROL KO, and TROL OX plants we aimed to further investigate the role of the TROL in antioxidant plant protection, as well as the influence of the TROL–FNR interaction on redox homeostasis system. By employing TEM and immunogold labeling we determined that high-light exposed TROL OX plants showed a significant increase in GSH content in all tested compartments except in the cytosol, in comparison to the plants grown at physiological light conditions. These results indicate that in conditions when TROL–FNR stoichiometry is altered, e.g., when TROL is overly accumulated, not only is the local thylakoid-associated ROS scavenging altered, but the oxidative balance of the entire plant cell is perturbed. This is not surprising since the TROL has dual localization, in thylakoid membranes and in the inner chloroplast envelope [3] and might therefore be involved in ROS signaling which originates from chloroplasts. It is therefore reasonable to assume that the TROL–FNR interaction and the associated dynamic FNR release could represent the source elements in the redox homeostasis cascade. Such scenario has already been proposed in our initiating study [2] and herein presented results only strengthen our primary assumption.

To conclude, in this work we present further evidence that the dynamic TROL–FNR interaction is responsible for energy distribution, utilization, and dissipation in photosynthetic membranes in Arabidopsis and likely in many other vascular plants. We additionally present the evidence that TROL and its interacting partners are the elements involved in maintaining cellular redox homeostasis by triggering protecting detoxification mechanism and thus coping with excessive ROS propagation.

## Figures and Tables

**Table 1:** Liquid chromatography-tandem mass spectrometry (GeLC-MS/MS) result. Proteins detected by the applied GeLC-MS/MS method after FLAG/HA tandem-affinity purification of protein complexes containing TROL from TROL OX Arabidopsis plants are presented in the table. Only proteins found in the final elution and those still bound to the affinity matrix, which were detected in all three independent TAP/MS analyses, are shown.

Protein	Accession Number	MW	Organism
Rhodanese-like domain-containing protein 4, chloroplastic OS = Arabidopsis thaliana OX = 3702 GN = STR4 PE = 1 SV = 2	sp|Q9M158|STR4_ARATH	49 kDa	*Arabidopsis thaliana*
Chlorophyll a-b binding protein CP26, chloroplastic OS = Arabidopsis thaliana OX = 3702 GN = LHCB5 PE = 1 SV = 1	sp|Q9XF89|CB5_ARATH (+1)	30 kDa	*Arabidopsis thaliana*
Ferredoxin--NADP reductase, leaf isozyme 1, chloroplastic OS = Arabidopsis thaliana OX = 3702 GN = LFNR1 PE = 1 SV = 1	sp|Q9FKW6|FNRL1_ARATH (+1)	40 kDa	*Arabidopsis thaliana*
Ferredoxin--NADP reductase, leaf isozyme 2, chloroplastic OS = Arabidopsis thaliana OX = 3702 GN = LFNR2 PE = 1 SV = 1	sp|Q8W493|FNRL2_ARATH (+1)	41 kDa	*Arabidopsis thaliana*
Lipoxygenase 2, chloroplastic OS = Arabidopsis thaliana OX = 3702 GN = LOX2 PE = 1 SV = 1	sp|P38418|LOX2_ARATH	102 kDa	*Arabidopsis thaliana*
PSAF OS = Arabidopsis thaliana OX = 3702 GN = AXX17_At1g31980 PE = 4 SV = 1	tr|A0A178WB32|A0A178WB32_ARATH	24 kDa	*Arabidopsis thaliana*
Chlorophyll a-b binding protein 1, chloroplastic OS = Arabidopsis thaliana OX = 3702 GN = LHCB1.3 PE = 1 SV = 1	sp|P04778|CB1C_ARATH (+5)	28 kDa	*Arabidopsis thaliana*
Chlorophyll a-b binding protein CP29.1, chloroplastic OS = Arabidopsis thaliana OX = 3702 GN = LHCB4.1 PE = 1 SV = 1	sp|Q07473|CB4A_ARATH (+2)	31 kDa	*Arabidopsis thaliana*
L-ascorbate peroxidase T, chloroplastic OS = Arabidopsis thaliana OX = 3702 GN = APXT PE = 2 SV = 2	sp|Q42593|APXT_ARATH (+1)	46 kDa	*Arabidopsis thaliana*
Photosystem II D2 protein OS = Arabidopsis thaliana OX = 3702 GN = psbD PE = 1 SV = 3	sp|P56761|PSBD_ARATH (+1)	40 kDa	*Arabidopsis thaliana*
Chlorophyll a-b binding protein 4, chloroplastic OS = Arabidopsis thaliana OX = 3702 GN = LHCA4 PE = 1 SV = 1	sp|P27521|CA4_ARATH (+1)	28 kDa	*Arabidopsis thaliana*
Protein plastid transcriptionally active 16, chloroplastic OS = Arabidopsis thaliana OX = 3702 GN = PTAC16 PE = 1 SV = 1	sp|Q9STF2|PTA16_ARATH (+1)	54 kDa	*Arabidopsis thaliana*
Uncharacterized protein OS = Arabidopsis thaliana OX = 3702 GN = AXX17_At3g54180 PE = 4 SV = 1	tr|A0A178VC12|A0A178VC12_ARATH (+1)	74 kDa	unknown
CaS OS = Arabidopsis thaliana OX = 3702 GN = AXX17_At5g22580 PE = 4 SV = 1	tr|A0A178UBZ3|A0A178UBZ3_ARATH	41 kDa	*Arabidopsis thaliana*
Chlorophyll a-b binding protein, chloroplastic OS = Arabidopsis thaliana OX = 3702 GN = AXX17_At1g16590 PE = 3 SV = 1	tr|A0A178WK60|A0A178WK60_ARATH	27 kDa	*Arabidopsis thaliana*
Photosystem II CP43 reaction center protein OS = Arabidopsis thaliana OX = 3702 GN = psbC PE = 1 SV = 3	sp|P56778|PSBC_ARATH (+1)	52 kDa	*Arabidopsis thaliana*
Uncharacterized protein OS = Arabidopsis thaliana OX = 3702 GN = AXX17_At1g65710 PE = 4 SV = 1	tr|A0A178W9Y8|A0A178W9Y8_ARATH (+1)	32 kDa	*Arabidopsis thaliana*
Photosystem I chlorophyll a/b-binding protein 3-1, chloroplastic OS = Arabidopsis thaliana OX = 3702 GN = LHCA3 PE = 1 SV = 1	sp|Q9SY97|LHCA3_ARATH (+1)	29 kDa	*Arabidopsis thaliana*
FtsH extracellular protease family OS = Arabidopsis thaliana OX = 3702 GN = VAR2 PE = 3 SV = 1	tr|A0A1P8AXC1|A0A1P8AXC1_ARATH (+1)	75 kDa	unknown
APE1 OS = Arabidopsis thaliana OX = 3702 GN = AXX17_At5g36010 PE = 1 SV = 1	tr|A0A178US93|A0A178US93_ARATH (+3)	31 kDa	unknown
ATP-dependent zinc metalloprotease FTSH 1, chloroplastic OS = Arabidopsis thaliana OX = 3702 GN = FTSH1 PE = 1 SV = 2	sp|Q39102|FTSH1_ARATH (+1)	77 kDa	*Arabidopsis thaliana*
Photosystem II 10 kDa polypeptide, chloroplastic OS = Arabidopsis thaliana OX = 3702 GN = PSBR PE = 1 SV = 1	sp|P27202|PSBR_ARATH (+2)	15 kDa	*Arabidopsis thaliana*
Cytochrome b559 subunit alpha OS = Arabidopsis thaliana OX = 3702 GN = psbE PE = 1 SV = 4	sp|P56779|PSBE_ARATH	9 kDa	*Arabidopsis thaliana*
Photosystem II CP47 reaction center protein OS = Arabidopsis thaliana OX = 3702 GN = psbB PE = 1 SV = 1	sp|P56777|PSBB_ARATH (+1)	56 kDa	*Arabidopsis thaliana*
Photosystem I P700 chlorophyll a apoprotein A2 OS = Arabidopsis thaliana OX = 3702 GN = psaB PE = 3 SV = 1	sp|P56767|PSAB_ARATH (+1)	82 kDa	*Arabidopsis thaliana*
HCF244 OS = Arabidopsis thaliana OX = 3702 GN = AXX17_At4g40280 PE = 1 SV = 1	tr|A0A178UXV2|A0A178UXV2_ARATH (+1)	44 kDa	unknown
Cytochrome b6 OS = Arabidopsis thaliana OX = 3702 GN = petB PE = 1 SV = 1	sp|P56773|CYB6_ARATH	24 kDa	*Arabidopsis thaliana*
Photosystem II protein D1 OS = Arabidopsis thaliana OX = 3702 GN = psbA PE = 1 SV = 2	sp|P83755|PSBA_ARATH (+1)	39 kDa	*Arabidopsis thaliana*
Photosystem I reaction center subunit V, chloroplastic OS = Arabidopsis thaliana OX = 3702 GN = PSAG PE = 1 SV = 1	sp|Q9S7N7|PSAG_ARATH (+1)	17 kDa	*Arabidopsis thaliana*
UPF0603 protein At1g54780, chloroplastic OS = Arabidopsis thaliana OX = 3702 GN = At1g54780 PE = 1 SV = 1	sp|Q9ZVL6|U603_ARATH (+1)	31 kDa	*Arabidopsis thaliana*
Photosystem I chlorophyll a/b-binding protein 2, chloroplastic OS = Arabidopsis thaliana OX = 3702 GN = LHCA2 PE = 1 SV = 1	sp|Q9SYW8|LHCA2_ARATH (+1)	28 kDa	*Arabidopsis thaliana*
Chlorophyll a-b binding protein 6, chloroplastic OS = Arabidopsis thaliana OX = 3702 GN = LHCA1 PE = 1 SV = 1	sp|Q01667|CAB6_ARATH (+2)	26 kDa	*Arabidopsis thaliana*
Photosystem I P700 chlorophyll a apoprotein A1 OS = Arabidopsis thaliana OX = 3702 GN = psaA PE = 2 SV = 1	sp|P56766|PSAA_ARATH (+1)	83 kDa	*Arabidopsis thaliana*
Chlorophyll a-b binding protein CP29.2, chloroplastic OS = Arabidopsis thaliana OX = 3702 GN = LHCB4.2 PE = 1 SV = 1	sp|Q9XF88|CB4B_ARATH	31 kDa	*Arabidopsis thaliana*
Chlorophyll synthase, chloroplastic OS = Arabidopsis thaliana OX = 3702 GN = CHLG PE = 2 SV = 1	sp|Q38833|CHLG_ARATH (+1)	42 kDa	*Arabidopsis thaliana*
Photosystem I reaction center subunit N, chloroplastic OS = Arabidopsis thaliana OX = 3702 GN = PSAN PE = 1 SV = 2	sp|P49107|PSAN_ARATH (+1)	18 kDa	*Arabidopsis thaliana*
GTP binding Elongation factor Tu family protein OS = Arabidopsis thaliana OX = 3702 GN = At1g07930 PE = 1 SV = 1	tr|F4HUA0|F4HUA0_ARATH	41 kDa	*Arabidopsis thaliana*
Photosystem I subunit O OS = Arabidopsis thaliana OX = 3702 GN = PSAO PE = 1 SV = 1	sp|Q949Q5|PSAO_ARATH (+1)	15 kDa	*Arabidopsis thaliana*
Probable plastid-lipid-associated protein 8, chloroplastic OS = Arabidopsis thaliana OX = 3702 GN = PAP8 PE = 1 SV = 1	sp|Q941D3|PAP8_ARATH (+1)	26 kDa	*Arabidopsis thaliana*
ATP synthase subunit alpha, chloroplastic OS = Arabidopsis thaliana OX = 3702 GN = atpA PE = 1 SV = 1	sp|P56757|ATPA_ARATH (+1)	55 kDa	*Arabidopsis thaliana*
Light-regulated protein 1, chloroplastic OS = Arabidopsis thaliana OX = 3702 GN = LIR1 PE = 1 SV = 1	sp|Q96500|LIRP1_ARATH (+1)	15 kDa	*Arabidopsis thaliana*
Chlorophyll a-b binding protein 3, chloroplastic OS = Arabidopsis thaliana OX = 3702 GN = LHCB3 PE = 1 SV = 1	sp|Q9S7M0|CB3_ARATH (+1)	29 kDa	*Arabidopsis thaliana*
At5g08050/F13G24_250 OS = Arabidopsis thaliana OX = 3702 PE = 2 SV = 1	tr|Q8VYV1|Q8VYV1_ARATH	17 kDa	*Arabidopsis thaliana*
Protein CURVATURE THYLAKOID 1A, chloroplastic OS = Arabidopsis thaliana OX = 3702 GN = CURT1A PE = 1 SV = 1	sp|O04616|CUT1A_ARATH (+1)	18 kDa	*Arabidopsis thaliana*
PGR5-like protein 1A, chloroplastic OS = Arabidopsis thaliana OX = 3702 GN = PGRL1A PE = 1 SV = 1	sp|Q8H112|PGL1A_ARATH (+1)	36 kDa	*Arabidopsis thaliana*
Photosystem I reaction center subunit II-1, chloroplastic OS = Arabidopsis thaliana OX = 3702 GN = psaD1 PE = 1 SV = 1	sp|Q9S7H1|PSAD1_ARATH (+3)	23 kDa	*Arabidopsis thaliana*
Actin-7 OS = Arabidopsis thaliana OX = 3702 GN = ACT7 PE = 1 SV = 1	sp|P53492|ACT7_ARATH (+1)	42 kDa	unknown
AT4g28750 OS = Arabidopsis thaliana OX = 3702 GN = At4g28750/F16A16_140 PE = 2 SV = 1	tr|Q7FY22|Q7FY22_ARATH (+1)	12 kDa	*Arabidopsis thaliana*
Photosystem I iron-sulfur center OS = Arabidopsis thaliana OX = 3702 GN = psaC PE = 3 SV = 2	sp|P62090|PSAC_ARATH (+1)	9 kDa	*Arabidopsis thaliana*
Probable histone H2A variant 3 OS = Arabidopsis thaliana OX = 3702 GN = At1g52740 PE = 1 SV = 1	sp|Q9C944|H2AV3_ARATH (+1)	14 kDa	*Arabidopsis thaliana*
Tubulin beta chain OS = Arabidopsis thaliana OX = 3702 GN = AXX17_At5g62240 PE = 3 SV = 1	tr|A0A178ULE0|A0A178ULE0_ARATH	51 kDa	unknown

## Data Availability

The data presented in this study are available on request from the corresponding author. The data are not publicly available due to contract obligations by the Grant providers.

## Abbreviations

TROL	thylakoid rhodanase-like protein
TROL OX	Arabidopsis line overproducing TROL protein
TROL KO	Arabidopsis line not producing TROL protein, *trol*
FNR	ferredoxin: NADP^+^ oxidoreductase
RHO	rhodanase-like domain of TROL
PEPE	Pro-Val-Pro repeat-rich region of TROL
ITEP	highly conserved module of TROL necessary for establishing high-affinity interaction with FNR
Tic62	62 kDa protein component of the translocon at the inner envelope of chloroplasts
tAPX	thylakoidal ascorbate peroxidase
SOD	superoxide dismutase
GSH	glutathione
